# Triboelectric Nanogenerators as Power Sources for Chemical Sensors and Biosensors

**DOI:** 10.1021/acsomega.2c06335

**Published:** 2022-11-29

**Authors:** Gaurav Khandelwal, Swati Deswal, Ravinder Dahiya

**Affiliations:** † Bendable Electronics and Sensing Technologies Group, University of Glasgow, Glasgow G12 8QQ, U.K.; ‡ Bendable Electronics and Sustainable Technologies Group, Electrical and Computer Engineering Department, Northeastern University, Boston, Massachusetts 02115, United States

## Abstract

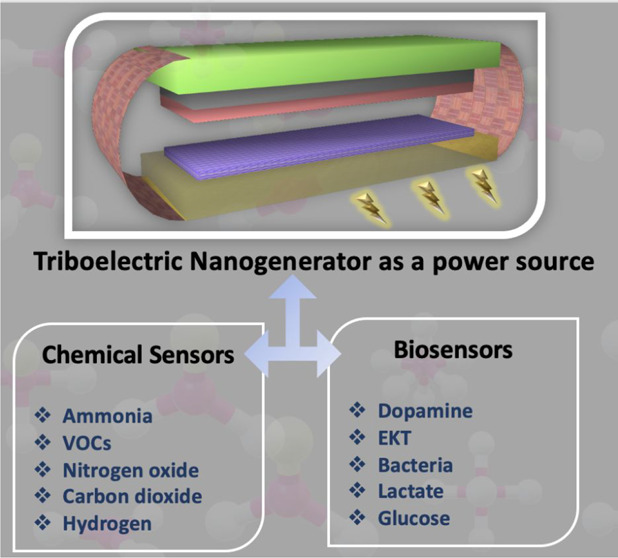

The recent advances of portable sensors in flexible and wearable form factors are drawing increasing attention worldwide owing to their requirement applications ranging from health monitoring to environment monitoring. While portability is critical for these applications, real-time data gathering also requires a reliable power supply—which is largely met with batteries. Besides the need for regular charging, the use of toxic chemicals in batteries makes it difficult to rely on them, and as a result different types of energy harvesters have been explored in recent years. Among these, triboelectric nanogenerators (TENGs) provide a promising platform for harnessing ambient energy and converting it into usable electric signals. The ease of fabrication and possibility to develop TENGs with a diverse range of easily available materials also make them attractive. This review focuses on the TENG technology and its efficient use as a power source for various types of chemical sensors and biosensors. The paper describes the underlying mechanism, various modes of working of TENGs, and representative examples of their utilization as power sources for sensing a multitude of analytes. The challenges associated with their adoption for commercial solutions are also discussed to stimulate further advances and innovations.

## 1 Introduction

The advancement in micro/nanotechnology has significantly enriched the quality and reliability of miniaturized sensors needed across multiple sectors including health monitoring,^1,2^ agriculture,^3^ aquaculture,^4^ environment monitoring,^5^ robotics,^6−8^ rehabilitation,^9,10^ automation,^11^ space,^12,13^ etc. Complemented by recent progress in flexible and printed electronics, these solutions continue to enrich the above areas and open new opportunities by allowing the detection of various chemical and biological analytes or parameters such as dopamine,^14^ glucose,^15^ tyrosine,^16^ and creatinine^17^ for human healthcare.^16,18^ The rapidly aging society and new forms of lethal diseases have pushed healthcare systems to adopt innovative solutions such as digital healthcare, which have contributed immensely to the rising demand for portable and wearable chemical sensors and biosensors globally.^19−21^ Numerous selective and sensitive chemical sensors and biosensors have been reported in the literature^22−30^ using distinct working mechanisms and exploiting the optical, piezoelectric, electrochemical, and enzymatic properties of various sensing materials [e.g., metal–organic frameworks (MOFs), two-dimensional (2D) materials, conducting polymers, carbon nanotubes (CNTs)].^31−34^ However, one of the long-standing problems with these sensors is the lack of a suitable source of power. Currently, batteries are most widely employed as the power source for these sensors. Batteries are difficult to recycle, offer a limited lifetime or require frequent charging, and involve toxic contents.^35,36^ As a result, different energy harvesting methods and self-powered sensors have been attracting attention.^37,38^

**Figure 1:**
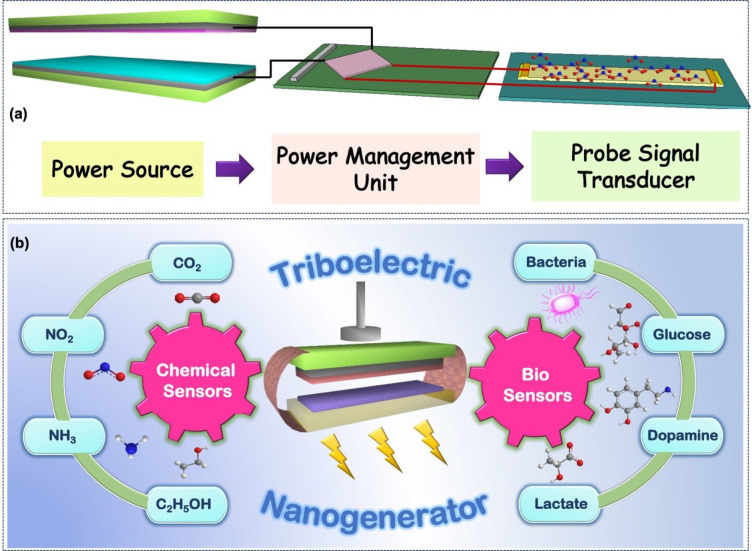
(a) Illustration of a sensor system powered by a TENG. (b) TENG as a power source for different chemical sensors and biosensors.

The mechanical energy harvesters based on piezoelectric and triboelectric effects offer a versatile solution as an alternative portable source of power.^39−45^ Among them, the triboelectric nanogenerator (TENG) presents unique advantages such as a wide choice of materials, distinct device working modes, light weight, high-power density, cost-effectiveness, and ease of fabrication. The broad range of triboelectric materials comprises natural materials, metals, metal oxides, 2D materials, conventional polymers, crystalline coordination polymers, ferroelectric materials, and textiles.^30,46,47^ Novel materials like MOFs and MXenes have been recently explored as the active layer for a TENG device.^25,48^ MOFs provide high specific surface area, tunable porosity and size, and ease of postsynthetic modifications. The MXene Ti_3_C_2_T_*x*_ exhibits triboelectric behavior similar to that of Teflon and can also be used as an electrode due to its high conductivity.^48^ The output performance of TENGs can be easily modulated by creating surface nano/microstructures, ion implantation, and chemical functionalization or modifications.^49−54^ However, TENGs must also meet a few standard requirements to power sensors effectively. This includes continuous and stable direct current (DC) output, adequate power to operate the sensors, and high stability in the working environment of the sensor. TENGs generally produce an alternating current (AC) output signal which needs to be processed using a power management unit (PMU), comprising a rectifier and energy storage device, as shown in Figure 1a. Apart from being a power source, TENGs can also act as active sensors where a chemical or biological analyte induces direct variation in the TENG output.^37,55^

This review presents a comprehensive description of the use of TENGs as the source of power for sensor systems. The TENG working modes are explained to understand their suitability for powering chemical sensors or biosensors along with the TENG figure of merits. Then, emphasis is placed on the TENG-powered chemical sensors for detection of ammonia, nitrogen dioxide, carbon dioxide, and volatile organic compounds (VOCs) (Figure 1b). This discussion is further expanded in section 4, where TENG-powered biosensors for detection of dopamine, bacteria, glucose, and lactate, etc. (Figure 1b) are presented. Finally, the summary and future perspective are presented to promote further innovations in TENG-enabled sensing.

## 2 TENG Working Principle and Device Modes

**Figure 2:**
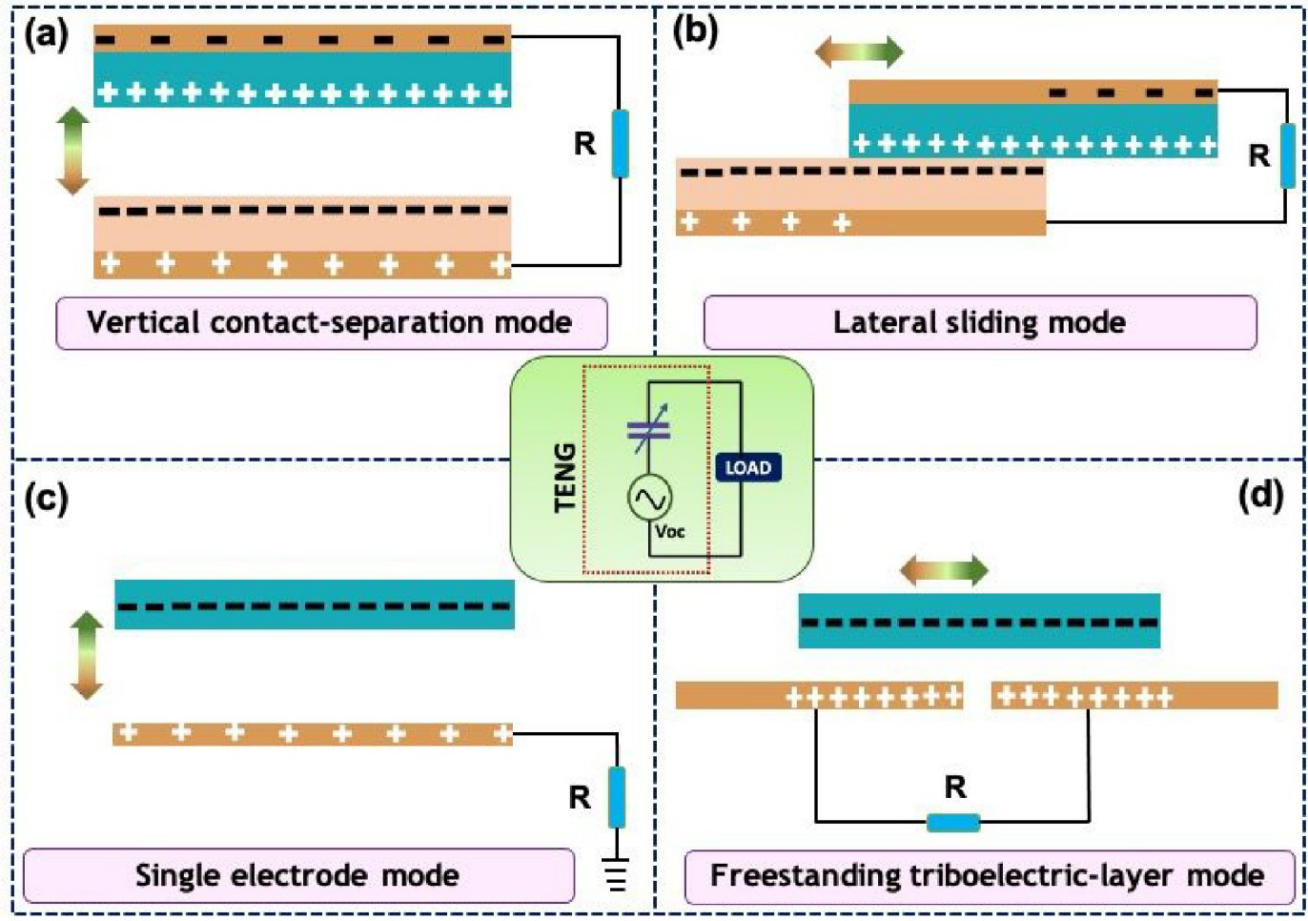
Four modes of TENG: (a) vertical C-S mode, (b) LS mode, (c) SE mode, and (d) FT layer mode.

A TENG converts mechanical energy into electricity via the coupling effect of contact electrification or triboelectrification and electrostatic induction.^53,56,57^ Contact electrification occurs when two dissimilar materials come in close contact with each other. Generally, two materials in frictional contact develop charges depending on their ability to gain or lose electrons. This information can be ascertained from the triboelectric series wherein materials are organized based on their propensity for gaining or losing electrons.^58^ Materials that lie far apart in the triboelectric series are always the preferred choice for fabrication of high-performing TENGs. Thus, the triboelectric series is often the starting point for selecting materials for TENGs. Compared to other energy harvesters such as piezoelectric nanogenerators (PENGs), TENGs produce high output, and they are comparatively easy to design. Further, they do not have a huge material restriction, offer design flexibility, and can be easily shaped in flexible and wearable form factors.^37,59−61^ Further, triboelectrification can also occur in solid–liquid contacts along with solid–solid interactions.^62^ A TENG can be considered as a capacitor with varying capacitance, as illustrated in the center of Figure 2, which displays the four working modes of TENG, viz., vertical contact-separation (C-S) mode, single-electrode (SE) mode, lateral sliding (LS) mode, and freestanding triboelectric layer (FT) mode.

### 2.1 Vertical Contact-Separation Mode

The vertical C-S mode is one of the simplest TENG modes, which offers high output, stability, and a facile design approach. The vertical C-S mode can either have dielectric-to-dielectric or dielectric-to-metal contact.^63^ The basic C-S mode is depicted in Figure 2a. It is composed of two triboelectric layers backed by metal electrodes. When two such materials contact each other upon the application of force, they develop equal and opposite charges. When the force is removed, the top active layer starts to move away from the bottom layer resulting in a potential drop. This potential drop is compensated by the flow of electrons from one electrode to the other, producing one-half cycle of the TENG’s AC output. An equilibrium state is achieved when the two layers are completely separated. In the presence of force, the top active layer begins to move toward the bottom layer, leading to the flow of electrons in the reverse direction, thus completing the full AC cycle of the TENG output. The periodic contact separation generates the TENG’s output.^63−65^

### 2.2 Lateral Sliding Mode

In the LS mode, both active layers are backed by electrodes. The relative sliding of one layer over the other creates a potential difference for the flow of electrons to produce an electrical output. As indicated in Figure 2b, the outward movement of the top layer relative to the bottom layer alters the contact area between them to create the charge separation. Thus, electrons flow from the bottom electrode to the top electrode until the top layer completely slides out. Similarly, inward movement of the top layer produces the flow of electrons in the reverse direction to complete the TENG’s AC output.^64,66^ This mode suffers exorbitantly from wear and tear, and thus several apparent attempts (e.g., using lubricants) have been made to alleviate this issue.^67^

### 2.3 Single-Electrode Mode

The SE mode can function in contact-separation as well as in sliding motion. The underlying working principle remains the same, i.e., the coupling effect of contact electrification and electrostatic induction. However, in single-electrode mode, one layer remains free to move and is devoid of any electrical connection (Figure 2c). The electrode connected to the ground serves as the reference for electric potential. In this mode, the contact separation or sliding of the movable layer creates a potential difference for the flow of electrons.^37,68^

### 2.4 Freestanding Triboelectric Layer Mode

The FT layer mode is easy to implement for practical applications as the moving layer does not have any electrode. Although the SE mode also presents the advantage of a free layer, the efficiency of the SE mode is much lower than that of the FT mode. Figure 2d shows the FT layer mode TENG, wherein two electrodes are separated by a small gap and are placed underneath the active triboelectric layer. The movement of a precharged triboelectric layer concerning electrodes creates an asymmetric charge distribution, leading to a potential difference. The generated potential difference is then balanced by the flow of electrons from one electrode to another, hence producing the TENG output.^69,70^ FT mode can operate in noncontact as well as in C-S.

The above-discussed four modes of TENGs can be compared using the established figures of merit (FOM). The TENG performance figure of merit (FOM_P_) entails structural (FOM_S_) and material (FOM_M_) figures of merit.^71^ FOM_M_ depends on the surface charge density (σ) of the material, and hence, materials with high σ can generate high output. The FOM_S_ for different working modes follows this trend: contact freestanding triboelectric (CFT) > C-S > sliding freestanding triboelectric layer (SFT) > LS > single-electrode contact mode (SEC).^71^ The CFT mode delivers the highest output and is, therefore, an ideal choice for development of power sources. However, the FT layer mode involves a complex structure and cumbersome fabrication process. Alternatively, C-S mode devices are easy to fabricate and offer high output characteristics in conjunction with excellent device stability.^54,64^ Moreover, the influence of environmental parameters such as humidity can be readily reduced in such devices by envisaging encapsulation techniques. The output of the TENG also varies with the applied force and frequency. With the increase in force, the effective or real contact area between the contact layer increases drastically, which leads to higher output.^53^ Similarly, with increased frequency, the flowing electrons reach equilibrium more swiftly leading to an increase in output with frequency.

## 3 TENGs as Power Sources for Chemical Sensors

This section discusses TENGs as power sources for chemical sensors. When a TENG is used as a power source, its stability is the key requirement. The ambient conditions highly influence the output of TENGs. Therefore, fully packaged, temperature- and humidity-resistant devices have a higher potential for self-powered sensing applications. Most chemoresistive gas sensors require complex materials to attain high selectivity and sensitivity.^72^ The material synthesis generally involves toxic chemicals and solvents that are not always eco-friendly.^72^ The high output of a TENG can also be used to create gas discharge, which can be used for self-powered sensing. The gas-discharge-based sensors here offer an advantage over chemoresistive gas sensors as they do not require any complex material. The introduction of another gas in the discharge process alters the plasma formation and changes the characteristics of the discharge.

This section elaborates the use of TENGs as attractive power sources for ammonia (NH_3_), nitrogen oxides, VOCs, and hydrogen sensing along with the sensing mechanism. The effect of individual analytes on human health and the environment are described in each subsection.

### 3.1 Ammonia Sensor

**Figure 3:**
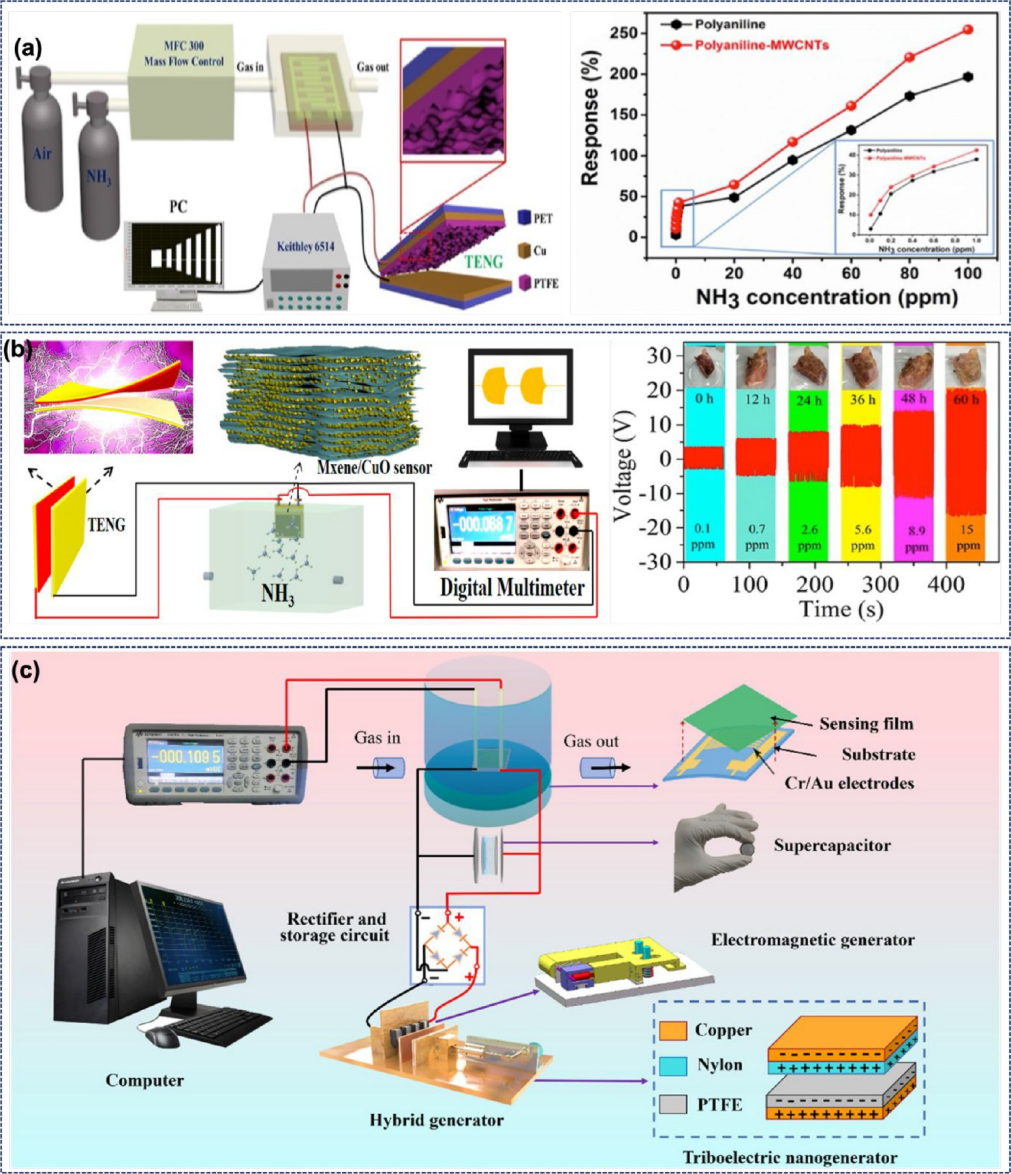
(a) Schematic illustration of the gas sensing setup and response of PANI- and PANI-MWCNTs-based sensors toward different ammonia concentrations. (Reprinted with permission from ref (73). Copyright 2018 Elsevier.) (b) Concept of the TENG-driven self-powered ammonia sensor and variation in voltage due to the release of ammonia from pork stored for different times at room temperature. (Reprinted with permission from ref (74). Copyright 2021 American Chemical Society.) (c) Schematic of an ultralow-concentration PANI/MXene sensor driven by a supercapacitor charged using a TENG/EMG device. (Reprinted with permission from ref (75). Copyright 2021 Elsevier.)

Ammonia is a toxic air and water contaminant and can cause severe damage to human health. For example, NH_3_ is reported to induce nausea, headache, and pulmonary disorders and can even lead to death at high concentrations. Moreover, ammonia is a key biomarker for the detection of kidney diseases. Thus, the detection of ammonia is important for monitoring of humans’ health and well-being, and as a result, self-powered triboelectric ammonia sensors (TEASs) combining a TENG with sensors based on different materials [e.g., polyaniline-multiwall carbon nanotubes (PANI-MWCNTs)] have been reported.^73^Figure 3a illustrates the setup for the gas sensing measurements. Here, the TENG consists of surface-structured poly(tetrafluoroethylene) (PTFE) and Cu as the contact layer, where Cu plays the dual role. The sensor part comprises PANI-MWCNTs deposited on poly(ether imide) (PEI) with interdigital electrodes (IDTs). The TENG produced an output voltage and current of 83 V and 6.55 μA, respectively. The NH_3_ can change the PANI from emeraldine salt (ES) to emeraldine base (EB) by donating electrons to PANI. Additionally, the absorption of NH_3_ decreases the carrier concentration in the MWCNTs. As a result of these, the absorption of NH_3_ alters the resistance of the sensing material and the output of the TENG increases with the ammonia concentration. Figure 3a also shows the response of the sensor in the range of 0.01–100 ppm. The sensor showed a linear response between 0.2–1.0 and 20–100 ppm and also good ability under bending conditions. The TEAS shows the response and recovery times of 8 and 120 s for 0.6 ppm of NH_3_. The utility of this type of sensor was also demonstrated for practical application by developing an alert system and preliminary testing of the human breath for NH_3_. The observed response and recovery times for human breath were 32 and 100 s, respectively.^73^ Other examples of TENG-based ammonia sensors include the elastic sponge TENG (ES-TENG), which is composed of a PANI nanowire conducting sponge and PTFE active layers.^76^ In this case, the TENG is connected to the conducting PANI sponge sensing element. The ES-TENG produced an output voltage and current of 540 V and 6 μA, respectively. The sensor can work in the wide concentration range of 1–2400 ppm with a fast response time of just 3 s.^76^

Other recent examples of self-powered NH_3_ sensing via a TENG include the novel hybrid material Ti_3_C_2_T_*x*_ MXene and MOF-derived Cu.^74^Figure 3b shows the self-powered MXene/CuO sensor driven by a TENG. In this case, the TENG was fabricated using latex and PTFE active layers, and it produced an output voltage and current of 810 V and 34 μA, respectively. The MXene–CuO hybrid offers the highest surface area of 20.7 m^2^ g^–1^, and this is followed by MXene (15.3 m^2^ g^–1^) and MOF-derived CuO (6.8 m^2^ g^–1^). The working principle of this type of sensor is the same as explained above, i.e., change in resistance due to the interaction of NH_3_ with the functional groups present on the MXene, and CuO providing more interaction sites. The resistance of MXene/CuO increases with the ammonia concentration, and this leads to an increase in the TENG output. The sensor works in the linear range of 0–100 ppm with excellent response/recovery times of 45/29 s. The sensor was also tested to check the quality of pork, which releases NH_3_ gas as it rots.^77^Figure 3b shows the released NH_3_ concentration from pork measured by this sensor at different storage times in ambient conditions.^74^ Later, an electromagnetic–triboelectric hybrid generator was used to power the ammonia sensor via a supercapacitor.^75^ Here, PANI was polymerized on MXene to improve the sensor and supercapacitor performance and to prevent MXene restacking. The PANI/MXene (V_2_C) was used as an active sensing material in the sensor and as an anode in the supercapacitor. Figure 3c depicts the concept of self-powered ammonia sensing. The NH_3_ sensor has a fast response and recovery time of 9 s and can exhibit an excellent response of 14.9% at ultralow NH_3_ concentration (1 ppm). The PANI/MXene offers large adsorption sites for NH_3_, which reduces the connectivity of nanocomposites, leading to increased resistance. The resistance change of the sensor was also wirelessly transmitted via a Bluetooth module to a mobile device.^75^

**Figure 4:**
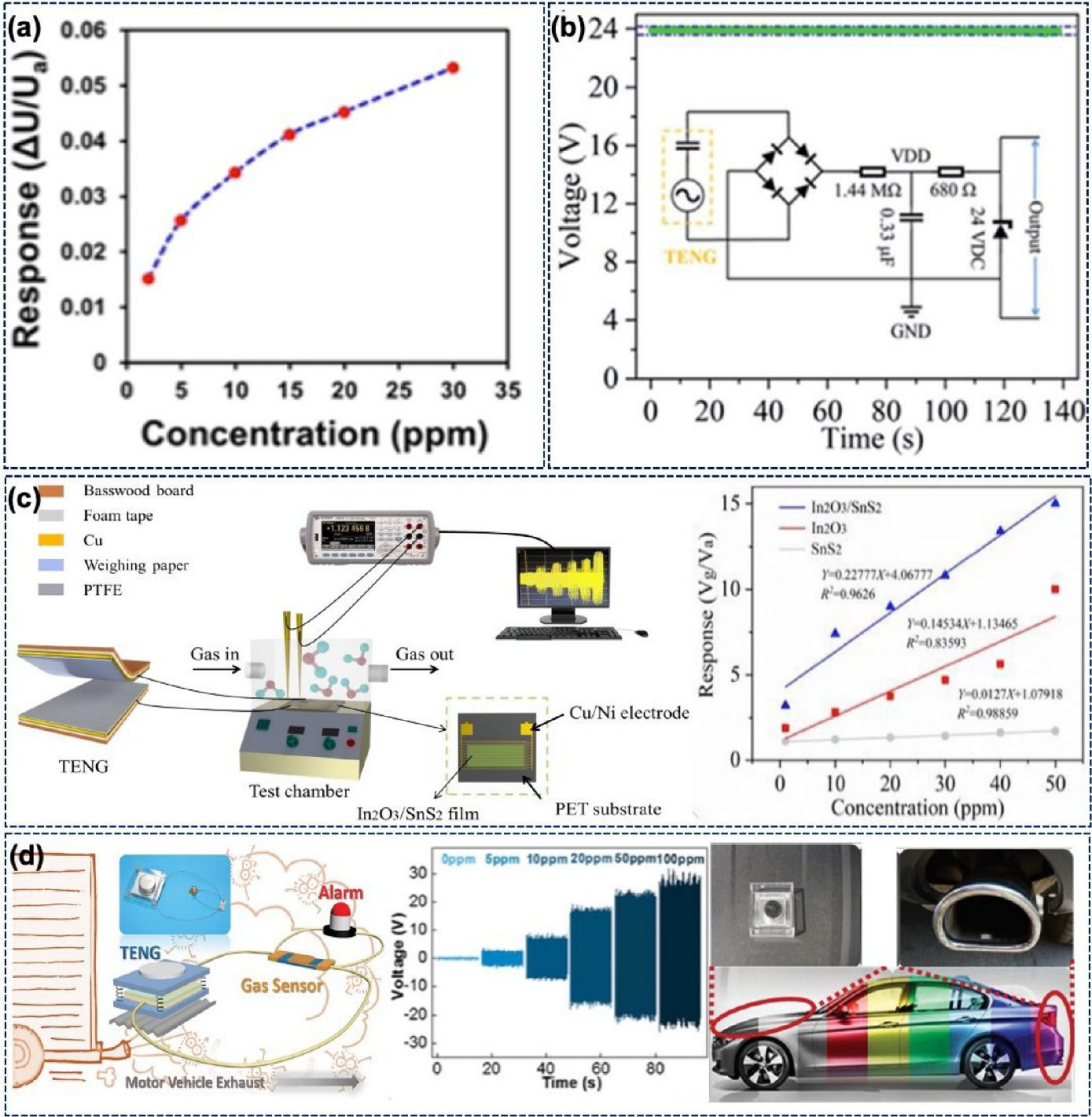
(a) Response of CNT-PPy-based self-powered sensors in the concentration range of 2–30 ppm ammonia. (Reprinted with permission from ref (78). Copyright 2022 Elsevier.) (b) A TENG is integrated with a rectifier circuit to obtain a constant output of 24 V to drive the sensors. (Reprinted with permission from ref (79). Copyright 2022 Royal Society of Chemistry.) (c) Schematic of the experimental setup for measurement of a TENG-driven NO_2_ sensor. (Reprinted with permission from ref (81). Copyright 2021 Royal Society of Chemistry.) (d) Schematic illustration depicting an automobile exhaust sensor driven by a TENG. Response of the sensor at different NO_2_ concentrations and the concept of harvesting the automobile vibration via the TENG to drive the sensor. (Reprinted with permission from ref (82). Copyright 2017 John Wiley and Sons.)

A recent example of a self-powered NH_3_ sensing system, developed with a TENG as a power source, used CNT-doped polypyrrole (PPy) (CNT-PPy) as the sensing element coupled to a signal collection and transmission unit.^78^ The system is designed for potential application in NH_3_-fueled ships where vibration from the engine can be used as mechanical excitation for the TENG. The five-layer TENG (V-TENG) used here can produce an output voltage of ∼350 V and a power density of 59.783 W m^–3^. CNT-PPy exhibits a p-type semiconductor behavior with hole conduction properties. In the presence of NH_3_, the holes are neutralized due to the electron transfer from NH_3_. Thus, adsorption of NH_3_ increases the resistance of the sensor via a decrease in charge carrier concentration. Figure 4a shows the response of the sensor in the concentration range of 2–30 ppm. The NH_3_ sensor exhibited a response time of 90 s and a low detection limit of 0.2 ppm with excellent selectivity and stability. The device was also integrated with Bluetooth for wireless signal transmission. The other related example is the gelatin–polyimide-based TENG (GP-TENG) which was used to drive a PANI/NiCo_2_O_4_-based ammonia sensor.^79^ The GP-TENG can produce a peak-to-peak voltage (Vpp) of 400 V. When a GP-TENG was integrated with a suitable rectifier circuit (Figure 4b), it was possible to have a constant output of 24 V to drive an ammonia sensor. The GP ammonia sensor is highly selective, with a response of 467% at 20 ppm ammonia. The sensor works on the basis of protonation and deprotonation of PANI. The sensor’s resistance increases in the presence of NH_3_, as under this condition the PANI changes to the EB state from the conducting ES state.

### 3.2 Nitrogen Oxide Sensor

Nitrogen oxide, generated from various sources, including fossil fuel combustion, automobiles, etc., is responsible for acid rain and reduced crop yields. A high level of nitrogen oxides can also lead to chronic lung diseases. Such harmful impacts of nitrogen oxide demand the development of portable and self-powered sensors. A few examples of such sensors include the UV-enhanced self-powered TENG-based sensor.^80^ In this case the TENG is composed of a PTFE negative layer, and Al serves as the electrode as well as the active layer. This work presented three types of gas sensors using reduced graphene oxide (rGO)–ZnO composite film, ZnO/rGO bilayer, and rGO/ZnO bilayer films. The sensing mechanism is similar to that of the other NO_2_ sensors, and the resistance increases from 0.28 to 0.70 MΩ when the nitrogen concentration increases from 0 to 100 ppm. The only difference is the generation of electron–hole pairs under UV light. The ZnO–rGO heterojunction increases the life of free electrons by restricting the combination of holes and photogenerated electrons. The rGO–ZnO composite exhibits the best response due to the p–n heterojunction. The number of available free carriers is higher for gas adsorption under UV, and therefore, it can be said that UV facilitates the high response in this case. The sensor was stable, selective, and showed the slow response/recovery times of 566/547 s. While the working of a standalone system was demonstrated using light-emitting diodes (LEDs), no experiments were conducted under sunlight to illustrate the applicability of the sensor.^80^

Figure 4c shows another TENG-driven NO_2_ sensor, where the TENG is connected in series with a chemoresistive In_2_O_3_ nanocubes/SnS_2_ nanoflower nanosensor.^81^ In this case, the TENG uses weighing paper and PTFE as the positive and negative triboelectric layers, respectively. The In_2_O_3_/SnS_2_ sensor is fabricated on a poly(ethylene terephthalate) (PET) substrate coated with the Cu/Ni IDT electrodes. Figure 4c also depicts the response of the sensor at different NO_2_ concentrations for SnS_2_, In_2_O_3_, and In_2_O_3_/SnS_2_. Among these, the In_2_O_3_/SnS_2_ sensor showed better response, linearity, and sensitivity. The resistance of In_2_O_3_/SnS_2_ varies between 100 kΩ and 1.38 MΩ, which is also the dynamic load matching region of the TENG, and therefore, the output of the TENG varies with the sensor’s resistance. The working mechanism of this sensor is also similar to that of the other NO_2_ sensor described above. In the presence of NO_2_, O_2_^–^ ions are absorbed and electrons get extracted. The number of electrons decreases with NO_2_ concentration, which eventually increases the resistance of the sensor. The In_2_O_3_/SnS_2_-based nanosensor showed response and recovery times of 45 and 147 s, respectively. Further, this sensor is highly selective and showed a stable performance over a 1 month period.^81^

The TENG-powered chemoresistive sensor can also find applications in vehicle emission testing systems (especially for NO_2_ detection).^82^ Automotive exhaust contains contaminants such as nitric oxides, carbon monoxide, and ammonia—all of which can add considerable pollutants to the air. An example of a self-powered sensing system for automotive applications is shown in Figure 4d. The metal–dielectric-type TENG used in this system generates energy from automobile engine vibrations. The TENG uses PTFE and Al as the active layers and produced a maximum output voltage and current of 75 V and 10 μA, respectively. The chemoresistive sensor is composed of an alumina substrate, Ag–Pd electrodes, and tungsten trioxide (WO_3_) as the sensing material. When exposed to NO_2_ gas, the sensor resistance can change from 68 kΩ to 10 MΩ. While the sensor is responding fast (response time 121 s), its recovery is slow (847 s). Nonetheless, the sensor is highly selective with poor response from interfering gases. When air is injected, the negative oxygen species, chemisorbed on the surface of WO_3_ nanorods, create a charge depletion layer. When the sensor is exposed to NO_2_, the NO_2_ molecules react with the absorbed oxygen species and capture electrons from WO_3_ to form intermediate NO_2_^–^ species. The NO_2_ interaction increases the depletion layer, which eventually leads to increased resistance. When connected with the TENG, the sensor acted as a variable resistor with resistance varying in the 68 kΩ to 10 MΩ range. Figure 4d shows the change in TENG output at different NO_2_ concentrations. The TENG output increases due to the rise in resistance of the sensor. The sensor showed full recovery after numerous cycles of gas injection and hence could be termed as reliable. Moreover, the sensor was tested 30 times a month at 5 ppm of NO_2_ to confirm the long-term stability. The self-powered vehicle emission sensor was demonstrated by turning three parallelly connected LEDs on and off. Here, a shaker was used to mimic the vehicle engine vibrations. Figure 4d illustrates the concept of the self-powered testing system. The TENG can be placed on the engine to harness the vibrational energy, and the sensor can be fitted in the vehicle’s exhaust pipe.^82^

**Figure 5:**
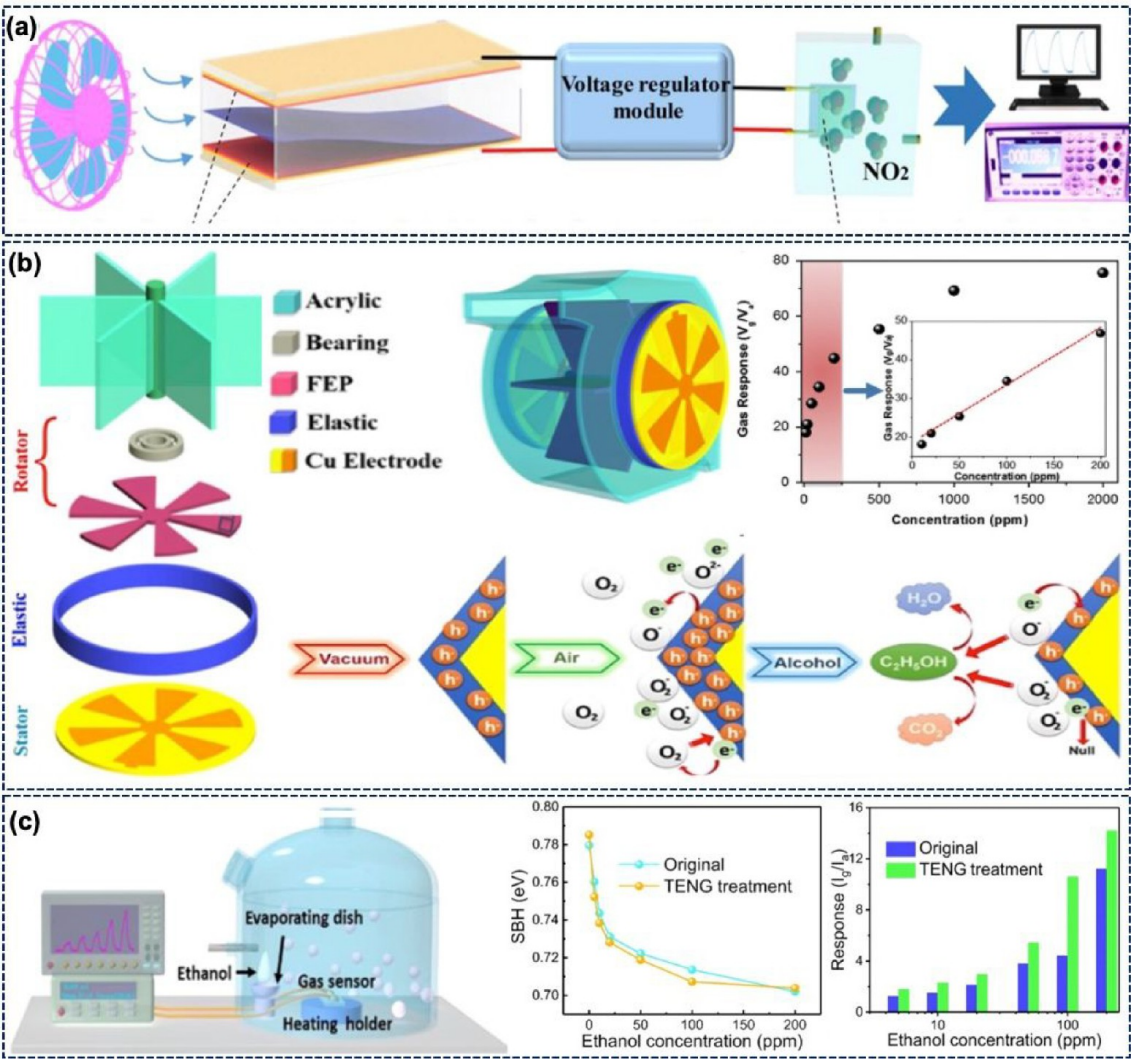
(a) Schematic illustration of a wind-driven TENG to power an NO_2_ sensor. (Reprinted with permission from ref (83). Copyright 2021 Elsevier.) (b) Design and mechanism of a blow-driven TENG for breath alcohol detection. Response of the sensor at different alcohol concentrations. (Adapted with permission from ref (86). Copyright 2015 Elsevier.) (c) Measurement setup, change in Schottky barrier height, and response of the sensor at different ethanol concentrations. (Adapted with permission from ref (87). Copyright 2020 American Chemical Society.)

Recently, a poly(vinyl alcohol) (PVA)/Ag nanofiber-based TENG has also been reported for powering a Ti_3_C_2_T_*x*_/WO_3_-based NO_2_ sensor.^83^ The concept of this self-powered wind-driven NO_2_ sensor system is shown in Figure 5a. Here, the PVA/Ag TENG produced an output voltage of 530 V and a current density of 359 mW m^–2^. The TENG-driven NO_2_ sensor exhibited a response of 510% at 50 ppm of NO_2_ gas, which was 15 times higher than the response of a resistive MXene/WO_3_ sensor. Moreover, with four TENGs, the Ti_3_C_2_T_*x*_/WO_3_ gas sensor can trace the source of NO_2_ in the wind. Due to the work function difference, the electrons move from MXene to WO_3_, leading to the formation of a Schottky junction at the Ti_3_C_2_T_*x*_ and WO_3_ interface. In the presence of NO_2_, the resistance of the sensor increases due to the capture of electrons from the conduction band, while MXene improves the gas adsorption and desorption rate.

### 3.3 Volatile Organic Compounds

Toxic VOCs such as benzene, toluene, acetone, methanol, ethanol, etc. directly influence human health. For example, benzene and toluene are carcinogenic; even in low concentration they may cause blurred vision, dizziness, and headache.^84^ Methanol, which is a widely used alcohol in numerous household agents, is toxic and can be harmful when absorbed, ingested, and inhaled.^85^ The wide use of VOCs makes their detection of utmost importance, and therefore, the field has attracted considerable interest.

An example of a self-powered system for detection of alcohol for a drunk driving test^86^ is shown in Figure 5b. The figure shows the design of a blow-driven TENG (BD-TENG)-based system for detection of alcohol in breath. The sensor is composed of a Co_3_O_4_ nanorod array as the alcohol adsorption sites. The resistance of this TENG-driven sensor changes during the absorption–desorption of alcohol. Figure 5b further depicts the reaction on the sensing element under the voltage applied by the BD-TENG. As Co_3_O_4_ is a p-type material, it has holes as majority charge carriers. On exposure to air, the Co_3_O_4_ is covered by the O^–^, O^2–^, and O_2_^–^ species, and a charge accumulation layer is formed near the surface. Due to the interaction of ethanol molecules and chemisorbed oxygen, a reduction of the charge carriers is observed in the accumulation layer in the presence of alcohol. This interaction leads to the release of free electrons and neutralizes the holes.^88−91^ As a result, the resistance of the sensor increases with the alcohol concentration. Figure 5b also shows the response of the sensor in the 10–2000 ppm alcohol concentration range. The device can work linearly in the low-concentration range. The device is selective and shows a maximum response to ethanol in comparison with other interfering gases. This BD-TENG was also combined with a wireless warning system which can switch between silent and panic states. Gas-sensitive materials such as tungsten oxide (WO_3_) have also been reported for ethanol detection.^92^ The ethanol-responsive changes in the resistance of the WO_3_ layer are in the variable region of the TENG load matching analysis. Also, the output of the device is shown on a liquid crystal display (LCD).

Another example of a TENG-based sensor is shown in Figure 5c, which depicts the experimental setup with a TENG connected to a methanol gas sensor via a rectifier. Here, the positive voltage of the TENG lowers the Schottky barrier height (SBH) of the ZnO nano/microwire (NMW)^87^ and enhances the sensor response by 139% (100 ppm ethanol). Figure 5c compares the SBH before and after the TENG voltage is applied and also depicts the response of the sensor in the original state and after combining it with the TENG. The TENG treatment also accelerated the recovery time but had a negligible effect on the response time. In the presence of air, the negatively charged oxygen species are chemisorbed on the ZnO NMW. The oxygen species are electron-withdrawing and increase the SBH of the sensor. As mentioned earlier, in the presence of ethanol, oxygen can react with the ethanol and lower the SBH. When the TENG is combined with the sensors, more oxygen molecules are absorbed, and hence, the depletion layer’s width can decrease. Therefore, more ethanol can interact with the oxygen molecules.

**Figure 6:**
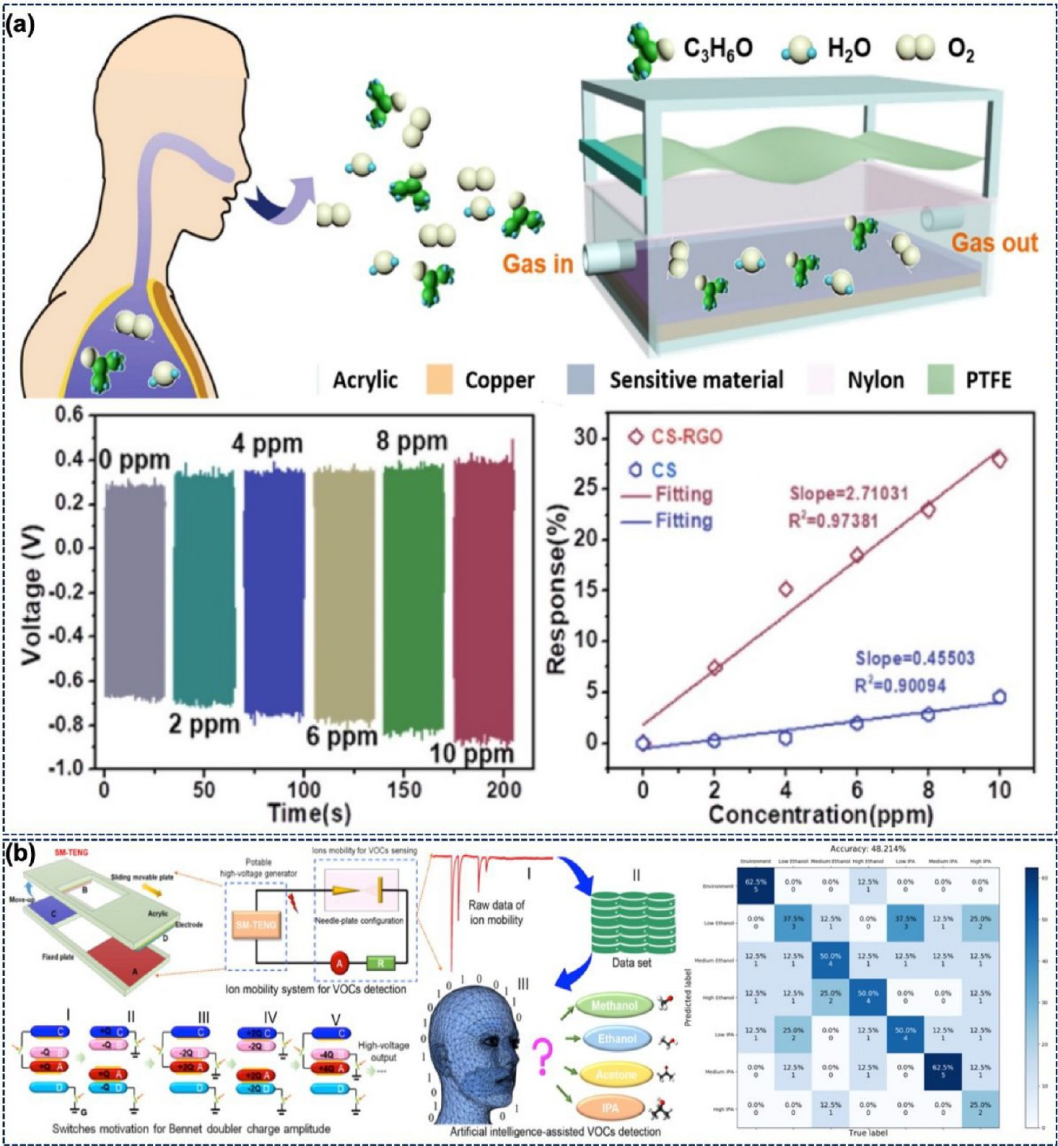
(a) Design of a WSAS for breath analysis. Variation in voltage and response at different acetone concentrations. (Adapted with permission from ref (93). Copyright 2020 Elsevier.) (b) ML-enhanced VOC sensor with an SM-TENG connected with an ion-mobility system for VOC detection. Results at different concentrations of ethanol and IPA enhanced by machine learning. (Reprinted with permission from ref (94). Copyright 2021 Elsevier.)

Figure 6a shows the chemisorption and contact electrification coupled to develop a self-powered acetone sensor (WSAS) for breath analysis.^93^ The TENG is composed of PTFE and nylon as the active layers. The PTFE vibrates and comes in contact with the nylon in the presence of airflow. The TENG-harvested energy is wirelessly transmitted to the metal electrode of the sensing layer. Here, the sensing layer is prepared by spray coating rGO–chitosan solution on a copper electrode. The TENG produced an output current of 345.6 nA at 5 Hz. The effect of the sensing distance on the wireless signal transmission confirms the 73% and 62% decline in the current and voltage, respectively, when the distance increases from 0.2 to 0.5 mm. Figure 6a shows the variation in voltage and response of the chitosan and CS–rGO films at different acetone concentrations. The response of CS–rGO is much better than that of CS due to more absorption sites and the area offered by the rGO. The sensor exhibited good selectivity with a 2-fold increase in response for the acetone compared to other toxic gases.

A light-enhanced VOC sensor using a WS_2_ microflake-based chemoresistive sensor is shown in Figure 6b. Here, the TENG with an output of 10 V and 15 μA at a frequency of 100 Hz has been used (instead of a DC power supply) to power the light source to enhance the sensor response.^95^ The Bennet-doubler-inspired TENG used here with machine learning (ML) led to an enhanced VOC sensor.^94^ Here, a multiswitched manipulated (SM) TENG serves as a power source for the ionizer. Moreover, the increase in TENG output with increase in contact area was also studied. The multiswitch was used to accumulate the charge on the electrode by operating in between “on” and “off” conditions. The SM-TENG produced an output voltage of ∼250 V and charge of ∼130 nC. Figure 6b also presents the ML-enhanced VOC sensor and the mechanism of the Bennet doubler. As the volume and weight of the ions influence the mobility pattern in the plasma discharge, the latter can be exploited to detect the VOCs. To improve further the electric field, this work also uses a needle–plate configuration. For VOC detection, the dark discharge is preferred over the glow and arc discharge due to the low power requirements and stable output. Figure 6b shows the recognition of the different concentrations of the isopropyl alcohol (IPA) and ethanol. The ML algorithm achieved an accuracy of 54.286% at a gap distance of 2 mm to classify the VOCs.

### 3.4 Carbon Dioxide Sensor

**Figure 7:**
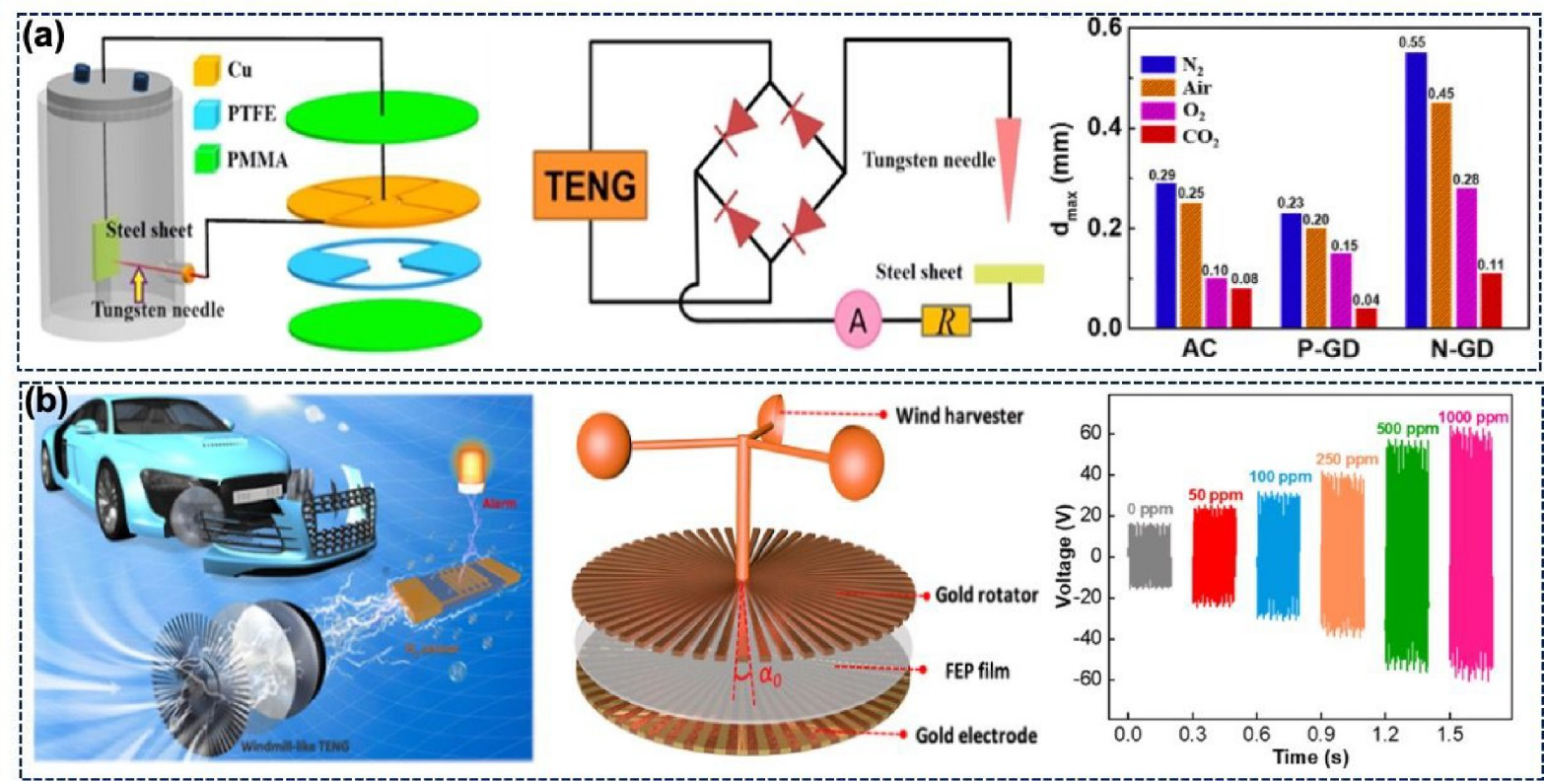
(a) Schematic illustration of a rotating TENG-driven gas-discharged-based sensor. Maximum distance (*d*_max_) between two electrodes in different ambient conditions under AC and negative and positive gas discharge. (Reprinted with permission from ref (72). Copyright 2018 Elsevier.) (b) Concept of hydrogen leakage detection in hydrogen fuel-powered vehicles. Design of a windmill-like rotating TENG and variation in the output voltage at different H_2_ concentrations. (Reprinted with permission from ref (96). Copyright 2021 Elsevier.)

In addition to being one of the major greenhouse gases, carbon dioxide (CO_2_) adversely affects human health by inducing nausea, headache, fatigue, respiratory inflammation, etc. TENGs can also be a sustainable power source for gas-discharge-based CO_2_ sensors.^72^Figure 7a depicts a gas-discharge-based sensor powered by a rotating TENG (R-TENG), which produced an output voltage of 1160 V across 1 GΩ. The gas-discharge system is composed of a tungsten needle and a stainless-steel plate. The gas breaks down at high voltage to produce a discharge consisting of electrons and positive ions. When CO_2_ is added to N_2_, the generated CO_2_^–^ ions can combine with positive N_2_ ions to repress the generation of the plasma. Thus, CO_2_ alters the discharge characteristics and increases the gas-discharge threshold voltage. The developed self-powered sensing system can be used for continuous detection, step mode, and threshold concentration detection. Figure 7a also shows the effect of the electrode gap distance on the AC and positive and negative gas discharge of N_2_, air, O_2_, and CO_2_. The results suggest that the threshold discharge voltage of CO_2_ is the highest. Moreover, the negative discharge (N-GD) can operate at low voltage and more effectively detect CO_2_. The threshold concentration can be detected as the gas discharge stops (stop phenomenon) when the CO_2_ concentration reaches the threshold value. The discharge current and frequency variation with the CO_2_ concentration can be used for the continuous and step detection modes. The step and continuous modes can be used when the CO_2_ concentration is below the threshold level.

### 3.5 Hydrogen Sensor

Hydrogen is a highly flammable gas which is currently used in applications such as hydrogen-powered vehicles and micro/nanofabrication. The safe storage of hydrogen is important, and as a result, different solutions have been explored. The examples include the Pd/ZnO nanorod-based hydrogen sensor powered by a windmill-like TENG (WL-TENG).^96^ The WL-TENG used in this work (Figure 7b) is designed to harvest wind energy when the vehicle is driven with hydrogen fuel. The impedance of the WL-TENG was tuned for the working region of the sensor by adjusting the angle of the windmill plate. This self-powered system has a TENG with a 3° plate angle serially connected with the Pd/ZnO H_2_ sensor. The output of the WL-TENG changes from 15 to ∼60 V in the hydrogen concentration range of 0–1000 ppm (Figure 7b). The sensor works on the basis of variation in the resistance of the Pd/ZnO sensing material. The Pd particles catalyze the H_2_ to active free radicals which then distribute on the ZnO nanorod surface and lead to a decrease in the resistance. The effect of H_2_ concentration was directly visualized via the number and brightness of the LEDs.^96^Table 1 summarizes the use of TENGs as power sources for the gas sensors.

**Table 1:** TENGs as Power Sources for Gas Sensors

device contact materials	device output	sensor	sensing material	range (ppm)	response time (s)	recovery time (s)	ref
PTFE–Cu	83 V, 6.55 μA	ammonia	PANI-MWCNT	0.01–100	89–103 (0.6–100 ppm)	120–127 (0.6–100 ppm)	(73)
NaNbO_3_/PDMS–Al	∼10 V, ∼15 μA	ammonia	WS_2_ microflake	5–60	252	648	(95)
PTFE–PANI NWs sponge	540 V, 6 μA	ammonia	conductive PANI sponge	1–2400	2	3	(76)
Ti_3_C_2_T_*x*_–MOF derived Cu	810 V, 34 μA	ammonia	MXene/CuO	0–100	45	29	(74)
PTFE–nylon	∼300 V	ammonia	PANI/MXene	0.3–10	9	9	(75)
PTFE ball–Cu	∼342 V, ∼26 μA	ammonia	CNTs–PPy	1–15	90	450	(78)
polyimide–gelatin	250 V, ∼50 μA	ammonia	PANI/NiCo_2_O_4_	0–20	25 (10 ppm)	59 (10 ppm)	(79)
PTFE–Cu	10 μA	CO_2_	gas-discharge based	1000–200000			(72)
FEP–Cu	∼18 V, ∼6 μA	ethanol	Co_3_O_4_	10–200	11	20	(86)
Kapton–Al	∼300 V	ethanol	ZnO NMW	5–200			(87)
FEP–Cu	∼35 V, ∼2 μA	ethanol	WO_3_	5–100			(92)
PTFE–Al	∼48 V, ∼15 μA	NO_2_	ZnO–rGO	20–100	566	547	(80)
PTFE–Al	∼75 V, ∼10 μA	NO_2_	WO_3_ NRs	5–100	121	847	(82)
PTFE–weighing paper	∼600 V, ∼60 μA	NO_2_	In_2_O_3_/SnS_2_	1–50	45	147	(81)
FEP–PVA/Ag	∼250 V, ∼2.5 μA	NO_2_	Ti_3_C_2_T_*x*_/WO_3_	0.5–50			(83)

## 4 TENGs as Power Sources for Biosensors

Biosensors are used to monitor the level of different biomarkers (glucose, dopamine, lactate, etc.), to detect different microbes, and for disease monitoring and drug discovery.^22,97^ The significant advancements in nanotechnology have led to the development of miniaturized sensors with low power consumption. The energy harvested by TENGs can be used to drive such sensors. The TENGs are able to meet different requirements to power the sensors including high energy conversion efficiency, resistance against sensor working conditions, and direct stable and tunable output.^98,99^ In this section, we discuss TENGs as power sources for detecting different analytes (dopamine, glucose, lactate), for enzyme detection, and to power electrokinetic trapping (EKT) in nano- or microfluidic devices.

### 4.1 Dopamine Sensor

**Figure 8:**
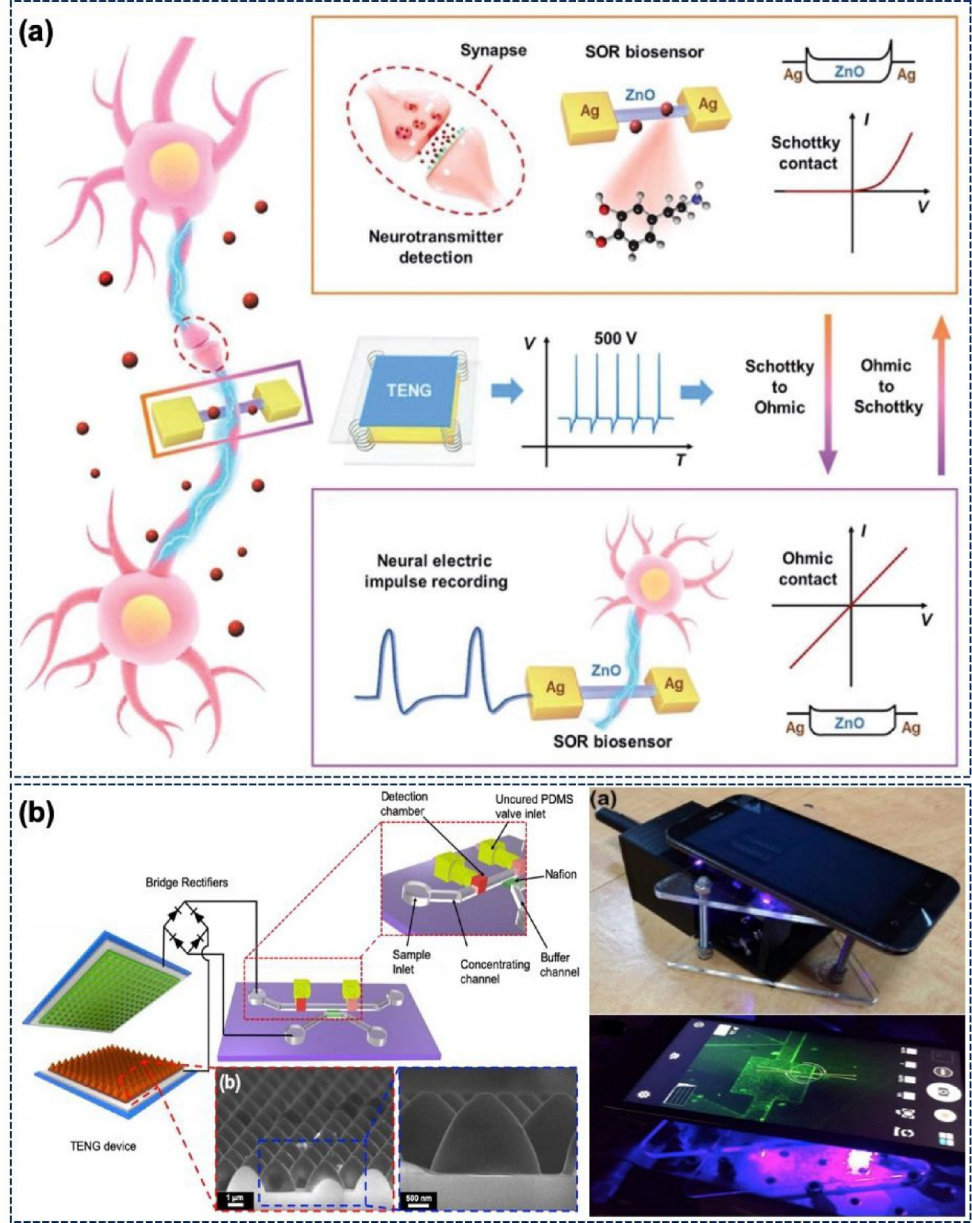
(a) Concept of highly sensitive neurotransmitter and neural electric impulse detection via a Schottky to an ohmic reversible biosensor. (Reprinted with permission from ref (105). Copyright 2019 John Wiley and Sons.) (b) Schematic illustration of a TENG-driven nanofluidic preconcentrator. Integration of a TENG-driven preconcentrator with a cell phone for monitoring an immunobead-filled channel. (Reprinted with permission from ref (106). Copyright 2019 Elsevier.)

Detecting nerve impulses and biomolecules (neurotransmitters) is critical in neuroscience for clinical diagnosis of diseases like Alzheimer’s, Parkinson’s, dementia, etc.^100,101^ Generally, Schottky contact and ohmic contact biosensors can be used to detect neurotransmitters and nerve impulses, respectively. Combining these two sensors in one tiny implantable device can be a boon to the clinical diagnosis of diseases related to levels of neurotransmitters (Alzheimer’s, Parkinson’s, etc.) and diseases related to the peripheral nerve conductions (multiple sclerosis, myotonia).^102−104^ One such sensor was developed using a TENG for reversible conversion of Schottky and ohmic contacts.^105^ The TENG is composed of aluminum and Kapton as active layers producing an output voltage of 500 V (before rectification) and 300 V (after rectification) and was used for the conversion of Schottky to ohmic contact. However, the high internal TENG impedance reduces the actual voltage on the biosensor to 20 V which was sufficient for effective conversion of contact. The Schottky to ohmic reversible (SOR) biosensor, shown in Figure 8a, can be used for the detection of the neural signal as well as the neurotransmitter. The initial Schottky contact of the ZnO nano/microwire can be used for sensitive and selective detection of dopamine. The Schottky contact transfers into the ohmic contact when treated with a voltage pulse from the TENG. The ohmic contact can be used for nerve signal detection. The ohmic contact changes back to Schottky contact when the voltage pulse is removed. The response of the Schottky contact biosensor for dopamine was 10 times higher than that of the ohmic contact. The ohmic contact biosensor fails to detect the lower dopamine concentrations. The Schottky contact biosensor demonstrated a sensitivity of 0.1 μmol mL^–1^. The ohmic contact worked excellently to detect the nerve signal when placed in the sciatic nerve trunk of a bullfrog. Moreover, the SOR biosensor was tested for simultaneous detection of dopamine and nerve signal before and after voltage treatment.

### 4.2 Nanofluidic Preconcentrator for Biosensing

Microfluidic devices are of significant importance in biomedical detection because they require a small sample volume. The detection of low-concentration molecules in microfluidic devices can be improved by EKT. However, the requirement of external DC voltage for EKT restricts the portability of microfluidic devices and development of point-of-care (POC) systems using them. In this regard, lightweight and miniaturized portable power sources are critical, and that makes TENGs relevant to such systems. An example of a TENG as power source to drive a nanofluidic preconcentrator for EKT of biomolecules is shown in Figure 8b, where a TENG is connected to a preconcentrator via a rectifier.^106^ A vertical C-S mode TENG with a peak–peak output of 50, 100, and 250 V operating at a frequency of 3.7 Hz is used in this work to trigger the nanofluidic preconcentrator. The intensity of the preconcentrator increases with the frequency of the device. The effect of higher frequency (7–37 Hz) was thus studied using the rotary TENG. A better concentration performance with the formation of two larger plugs was observed at a higher frequency. Finally, the TENG-based preconcentrator was integrated with a smartphone for immune sensing (Figure 8b). Figure 8b also depicts the image of the immunobeads-filled fluidic channel captured on a smartphone.

### 4.3 Bacterial Detection

**Figure 9:**
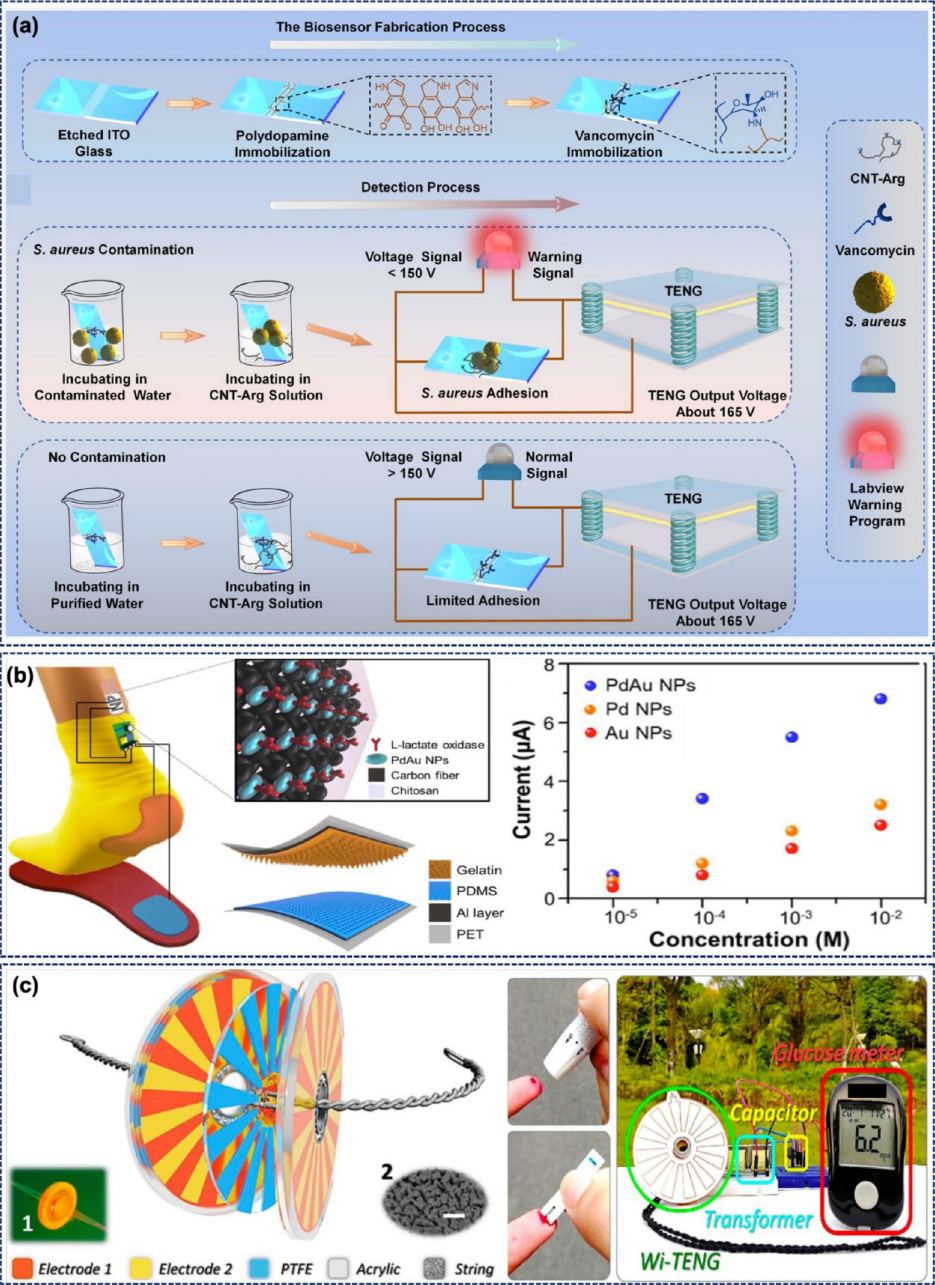
(a) Fabrication of a biosensor for selective capture of Gram-positive *S. aureus* bacteria and a schematic illustration of a TENG-driven *S. aureus* biosensing system. (Reprinted with permission from ref (107). Copyright 2021 Elsevier.) (b) Device design of a gelatin-based TENG for biomechanical energy harvesting to drive a lactate sensor. Variation in current at different lactate concentrations. (Reprinted with permission from ref (108). Copyright 2017 Elsevier.) (c) Design of a whirligig-inspired TENG and use of the harvested energy to power a glucose sensor. (Reprinted with permission from ref (109). Copyright 2018 Elsevier.)

Gram-positive bacteria like *Staphylococcus aureus* are pathogens that can cause food-borne diseases,^110^ and a simple and fast detection method is desired. Some of the existing methods such as polymerase chain reaction (PCR), enzyme-linked immunosorbent assays, and electrochemical impedance spectroscopy (EIS)^111−114^ are simple to use, but are also slow. Further, the need for an external power source affects their portability, and therefore, researchers have turned to TENGs. Figure 9a shows a proof-of-concept device using a vertical C-S TENG for bacterial detection in solution.^107^ A vertical C-S mode TENG was designed for the purpose with Al and fluorinated ethylene propylene (FEP) active layers. The TENG produced an output voltage of ∼165 V. This device uses the specific interaction between vancomycin and the bacterial cell wall for selective detection of Gram-positive bacteria (*S. aureus*). Moreover, a guanidine-functionalized MWCNT (CNT-Arg) is used for the signal amplification attributed to the high conductivity of MWCNTs. The specific interaction allows selective bacterial detection by reading the voltage variation of the sensor. Vancomycin has strong affinity toward a _(D)_-Ala-_(D)_-Ala peptide residue present on the bacterial cell wall. Guanidine functionalization helps in interacting with sulfates, carboxylic acid, and phosphates present on the bacterial cell membrane. The developed biosensing system successfully detected *S. aureus* with a limit detection value of 2 × 10 ^3^ CFU mL^–1^. Finally, the device was demonstrated as a warning system via a LabView program for the presence of *S. aureus* in contaminated water.^107^

### 4.4 Lactate Sensor

Lactate is involved in the anaerobic glycolytic pathways and can be produced in the brain, muscles, gut, and skin.^115^ A high lactate level can induce lactic acidosis,^116,117^ and it is also involved in several biochemical reactions during histogenic hypoxia, surgeries, sepsis, and respiratory failures,^117^ etc.—all of which make it important to monitor lactate levels. TENGs have been used in this area for the electrochemical synthesis of nanoparticles and lactate detection.^108^ The metallic nanoparticles were grown electrochemically on carbon fiber decorated with Pd–Au nanoparticles and modified with lactate oxidase to develop the anode of the sensing unit. The poly(dimethylsiloxane) (PDMS) and gelatin active layers based TENG used in this work produced an output voltage and current density of 500 V and 14 mA m^–2^. Figure 9b illustrates the concept of this self-powered system, which can also detect lactate in sweat. Figure 9b depicts the current variation across different lactate concentrations for Au, Pd, and PdAu nanoparticle based anodes. The sensor was highly selective for lactate compared to interfering species like creatinine, uric acid, and glucose.

### 4.5 Glucose Sensor

Glucose monitoring is important in the food industry, healthcare, and biotechnology. The monitoring of blood glucose levels is vital for diabetic patients.^118−120^ Many battery-powered commercial glucose meters are available worldwide, and TENGs can either replace or extend the battery lifetime for such systems. For example, the energy harvested by a TENG has been stored in a battery to power a glucose biosensor.^121^ Here, the TENG was fabricated by using Al foil and patterned PDMS layers, but other material combinations could be suitable too. The body motion energy was harnessed by placing the TENG between the clothes. The TENG produced an output voltage of 17 V and a current density of 0.22 μA cm^–2^ under normal walking. The battery could be charged to 800 mV in 2 h by tapping the clothes at a frequency of 2 Hz. Another example of TENG-powered glucose sensors is shown in Figure 9c. This system uses whirligig-inspired (wi) TENG,^109^ designed to produce a voltage, current, and charge of 153 V, 317 μA, and 310 μC, respectively. Figure 9c shows the use of a TENG for powering commercial glucose sensors. A 5 mF capacitor was charged to 3.5 V in 7.3 s using a wi-TENG with a power management circuit (PMC) to power the sensor.^109^Table 2 summarizes the use of TENGs as power sources for biosensors.

**Table 2:** TENGs as Power Sources for Biosensors

TENG mode	analyte	TENG output	ref
C-S mode	dopamine	500 V	(105)
C-S mode	immune sensing	50–200 V	(106)
C-S mode	bacteria	∼165 V	(107)
C-S mode	lactate	500 V, 14 mA m^–2^	(108)
C-S mode	glucose (commercial)	17 V, 0.22 μA cm^–2^	(121)

## 5 Summary and Future Perspectives

TENGs have witnessed remarkable advancement as an energy harvesting technology for myriad applications. Owing to the ease of fabrication, wide array of materials, low cost, and high output power, they are perceived as the front runners among various solutions for reliable power sources for chemical sensing and biosensing fostering healthcare transformation. This review provides key developments in self-powered chemical sensors, encompassing gas sensors and VOCs. It also sheds valuable insights into the deployment of TENGs as power sources for biosensors.

The existing TENGs can be used for the development of self-powered portable sensor systems, if a high level of device integration is achieved. The discontinuous AC output of TENGs makes them unsuitable for real-time applications as the majority of sensors require a DC power source. But TENGs are unique as they are compatible with most of the conventional common analysis methods which with further improvements can be used to develop novel self-powered sensing systems. To achieve TENG-based self-reliant sensing systems significant advancements are required in the following direction.

1. The continuous operation of the sensor necessitates stable output power from the TENG. A TENG produces discontinuous AC output, which cannot be used to power the sensors without an intermediate PMU. However, the currently used PMUs suffer from significant power loss and need significant advancement in terms of efficiency and miniaturization. Moreover, the printed circuit boards (PCBs) and breadboard used in designing PMUs are rigid and restrict the flexibility of the sensing system. Flexible sensing systems can be designed by using flexible printed circuits which are much easier to integrate for wearable applications.

2. As the underlying mechanism for TENG is built on surface charge density, the influence of environmental conditions such as humidity, temperature, and surface adsorption should be considered while devising TENGs for sensing applications. Encapsulating the device to prevent contamination and infiltration from the outer environment without compromising the efficiency is essentially required.

3. Since harvesting energy from the environment is subject to variations in the surroundings, which are time reliant and, in many instances, unpredictable, a high degree of integration is requisite for the portability of the sensors. Therefore, hybrid devices involving the integration of the TENG with a piezoelectric nanogenerator or an electromagnetic generator or a solar cell can ensure more reliable self-powered readouts. Nonetheless, the impedance mismatch among different energy harvesters must be addressed for efficient integration.

4. New computational methodologies based on artificial intelligence (AI) and its associated branches need significant attention for their application in self-powered sensors.^122^ The challenges associated in device design, output prediction, and device optimization can be addressed using AI or its associated branches. For sensors, AI can be used to recover information from the output signal (signal width, amplitude, and pattern), thus improving the data quality. AI tools can be used to predict the functional relationship between the device output and input parameters including environmental effects and the presence of analytes. In the future, ML algorithms or big data analysis can lead to the development of intelligent detection or sensor systems. Further, deep neural networks (DNNs) consisting of multiple interconnected artificial neurons can be used for quick prediction and analysis of self-powered sensor data.^123^

TENGs hold great potential in transforming the healthcare industry by acting as a sustainable power source for next-generation portable chemical sensors and biosensors. Further advances and improvements are expected to thrive in the years to come that will assist in defining the road toward the commercialization of sensors powered by triboelectrification.
